# Primary Progressive Aphasia Treatment: Current Treatment Options in Neurology Article Topic: Management of Primary Progressive Aphasia

**DOI:** 10.1007/s11940-025-00848-4

**Published:** 2025-09-20

**Authors:** Joshua G. Cahan, Borna Bonakdarpour

**Affiliations:** 1 Mesulam Institute for Cognitive Neurology and Alzheimer’s Disease, Feinberg School of Medicine, Northwestern University, Chicago, IL USA; 2 Ken and Ruth Davee Department of Neurology, Feinberg School of Medicine, Northwestern University, Chicago, IL USA; 3 Abrams Research Center on Neurogenomics, Feinberg School of Medicine, Northwestern University, Chicago, IL USA; 4 Kathryn Aring Piper Center for Frontotemporal Cognitive Disorders, Feinberg School of Medicine, Northwestern University, Chicago, IL USA

## Abstract

Primary progressive aphasia(PPA) is a rare neurodegenerative condition and variant presentation of Alzheimer’s disease or Frontotemporal Dementia. It is characterized by progressive decline isolated to language functions. PPA provides a model for understanding the anatomy of language, where each cortical language center corresponds to distinct PPA subtypes. Understanding this anatomy and its corresponding PPA subtypes helps clinicians choose testing, interpret imaging, and tailor treatment. These subtypes are termed agrammatic/nonfluent, semantic, and logopenic PPA. Each subtype is probabilistically associated with three proteinopathies: the amyloid and tau of Alzheimer’s disease and frontotemporal lobar degeneration due to Tau or TDP-43. We will discuss when biomarker testing is indicated and the nuances of choosing among the increasing array of biomarker tests to improve diagnostic certainty. While medical treatment is limited, there are increasing pharmacologic options for treating Alzheimer’s disease. Non-pharmacologic strategies can also be tailored to the patient’s specific subtype and caregivers’ needs.

## Introduction

Primary progressive aphasia(PPA) is a rare neurodegenerative syndrome and variant presentation of Alzheimer’s disease or Frontotemporal Dementia. Its incidence is around 0.56 persons per 100,000 person-years [1]. Prevalence among dementia patients is around 3% [2]. Partly due to its rarity, PPA poses a unique clinical challenge. This review provides a conceptual framework for understanding the various PPA syndromes through their neuroanatomical correlates to guide diagnostic workup and treatment strategies.

**Fig. 1:**
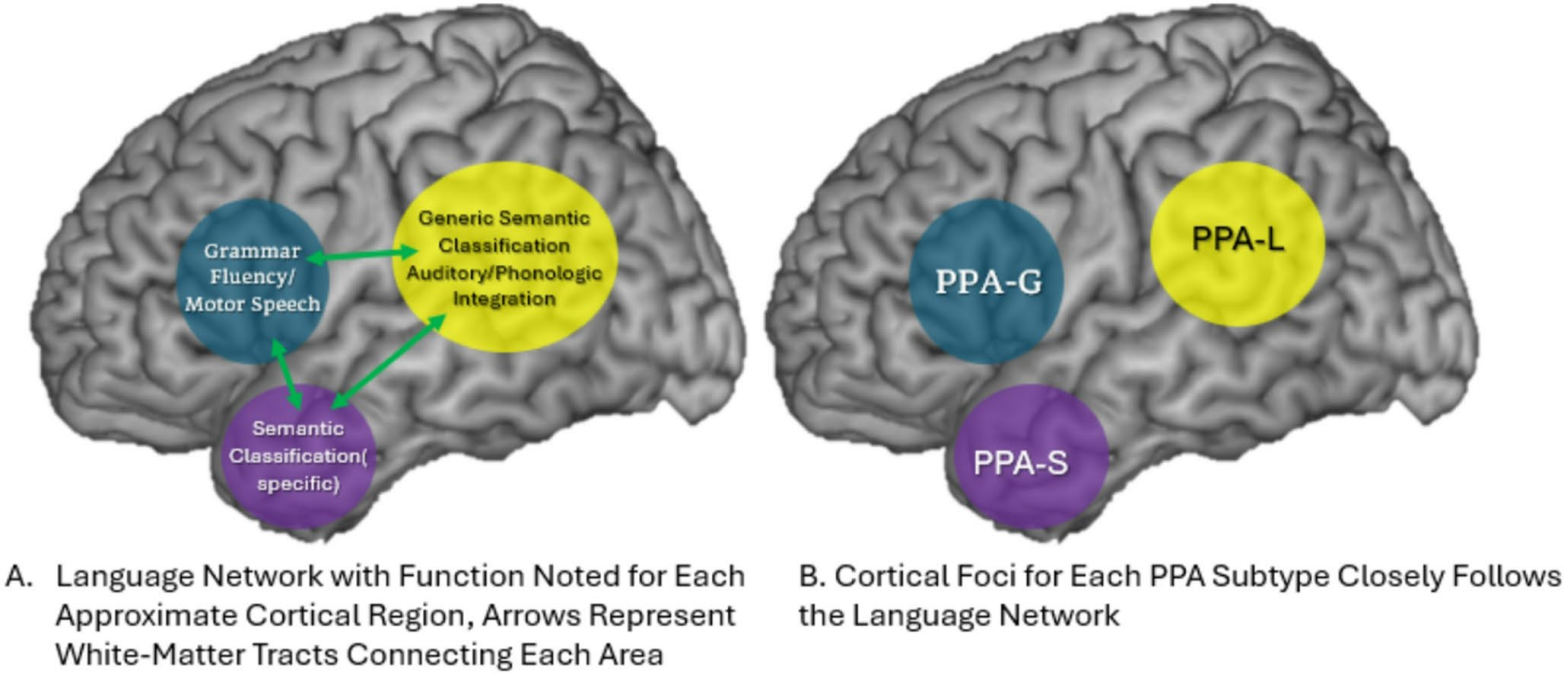
Neuroanatomy of language network and PPA syndromes

PPA is defined as a *progressive* deterioration in language, due to neurodegeneration, where language is the *primary* cognitive domain that is impacted. The aphasic syndromes caused by PPA are somewhat analogous but ultimately unique when compared to the more widely recognized stroke syndromes of a “Broca’s” and “Wernicke’s” aphasia. The novel speech/language syndromes are PPA-G(agrammatic variant, also known as the non-fluent variant), PPA-L (logopenic variant), and PPA-S (semantic variant) [3]. Each distinct PPA syndrome points to dysfunction in specific brain regions (Fig. 1) and a probabilistic association with underlying pathology. We will first address the neuroanatomical correlates of each clinical phenotype, followed by suggested workup to infer the underlying pathology, and finally, the treatment approach.

## Neuroanatomy of Language

Language consists of symbols used as a means of communication, most often in the form of spoken sounds. This is distinct from speech, which is a motor function. A dysarthria easily emerges from a non-dominant hemisphere stroke. Language function is supported by a group of brain regions connected by white matter tracts in the dominant hemisphere to form the language network, with speech intimately intertwined in the frontal lobe.

Language resides in the dominant hemisphere, typically the left (except for some left-handed individuals) [4]. Historically, two language epicenters are widely recognized. Discovered in the late 19th century, Paul Broca and later Karl Wernicke described distinct language deficits with vascular lesions in two different, now eponymous, brain regions [5, 6]. Expressive language difficulties with dysfluent speech and preserved comprehension characterize strokes in Broca’s Area(Brodman’s Area 44/45, pars opercularis/triangularis of the dominant frontal lobe). Contrastingly, comprehension deficits with incomprehensible albeit fluent “word-salad” characterize strokes in Wernicke’s Area(Brodman’s Area 22, the dominant superior temporal gyrus). In this classical representation, expressive language is supported by the frontal lobes and decoded in the temporal lobe. This understanding was advanced through Geschwind’s conceptualization of distributed neural networks. The *Wernicke-Geschwind* model incorporated white matter and disparate cortical anatomy, supported by curious disconnection syndromes such as alexia without agraphia [7–9]. 

Consequently, it was a protégé of Geschwind, Mesulam, who first described the syndrome of PPA [10]. In doing so, a new model for studying language biology was opened. By studying PPA-S, a third nexus of the language network underlying semantic information was identified in the dominant temporal pole. The temporal pole is important for assigning subordinate(specific) or superordinate(generic) categories to varying objects, with more processing required for more specific and unique words. Mesulam aptly summarizes the hierarchical demands of processing in object naming saying, “there are many exemplars that can be called ‘hat’, and many more that can be called ‘stuff’ so that the naming of an entity at a generic or nondescript level can be achieved with fewer neural resources.” Likewise “fedora” or “beret” are more specific and effortful to use appropriately. It is the temporal pole that feeds-forward signals to the temporoparietal junction, near Wernicke’s area, honing these categorical inferences in a top-down manner [11]. 

### The PPA Subtypes

#### Nonfluent Agrammatic PPA (PPA-G)

The agrammatic or non-fluent variant of PPA(PPA-G) is characterized by isolated, slowly progressive difficulty in sentence production. This may manifest as errors in the order of words in sentences, using the wrong article or tense. There may be difficulty understanding non-canonical grammar, such as “the cat that is being bitten by the dog” as opposed to “the dog chasing the cat” or, in more severe stages, individual grammatical morphemes [12–14]. There is often a mild anomia and there can be a reduced fluency in the number of words produced per minute. The anomia and loss of fluency are grossly similar to Broca’s aphasia, owing to a common left frontal localization. The grammar difficulty implicates the frontal lobes’ role in sequencing words and tracking relationships within sentences [14]. This and other features are unique when compared to aphasias caused by stroke. In PPA-G, naming may be worse for verbs than objects [15]. Writing is often impacted by similar errors. Often, problems in naming and fluency are from motor planning deficits in speech, referred to ask “speech apraxia”. With this deficit, patients may have abnormal length or stress of syllables when speaking. Repetition is challenging, particularly for complex multi-syllabic words, and speech may be challenged with phonemic paraphasic errors. This apraxia, in the absence of other PPA-G features, may be termed a “primary progressive apraxia of speech”. It also shares anatomic and etiologic features with PPA-G, often owing to L frontal dysfunction, typically related to FTLD-Tau [16, 17]. 

#### Semantic PPA (PPA-S)

The primary difficulty in PPA-S is naming and comprehension of single words. The initial symptoms may be quite mild without any problems in comprehension. As the disorder progresses and more semantic knowledge is lost, the anomia worsens, and word recognition deteriorates [13, 14]. Contrasting PPA-G, naming is worse for nouns compared to verbs [15]. Also, sentence and grammar comprehension remain intact until late in the disease. The dysfunction of PPA-S is anatomically centered in the dominant temporal pole, a nexus of the ventral language stream, causing loss of semantic information. This may cause “taxonomic interference” where words are named at more generic as opposed to specific levels, leading to semantic paraphasic errors(saying an incorrect word) in speech. Greater specificity in word choice requires greater mental resources. Thus, generic words are often substituted in place of more specific ones as the ability for semantic classification deteriorates. Likewise, at all stages in the disease, naming more specific, low-frequency, words is challenging compared to high frequency generic words. Reading can often be impacted, specifically for non-phonetically spelled words like “pint” or “choir”. With progression of disease and accompanying involvement of the non-dominant temporal lobe, patients may develop difficulty in face and object recognition. The combination of progressive semantic aphasia, impaired object recognition, and impaired face recognition (prosopagnosia) constitutes a designation of semantic dementia [18, 19]. 

#### Logopenic PPA (PPA-L)

PPA-L typically begins with subtle word-finding difficulty that slowly advances towards dysfluent verbal output with frequent word-finding pauses and circumlocutions. Like in PPA-S, less common words are most impacted. Unlike the PPA-G and PPA-L, grammar and comprehension are preserved. Object naming is impaired but less profoundly than in PPA-S. Early on, object naming may be preserved but other changes in speech like reduced number of words produced per minute or mean utterance length in spontaneous speech may be impacted. Oftentimes in PPA-L, repetition is impaired and is considered a core feature [20]. 

#### PPA-Plus (PPA+), Mixed, and Unclassifiable PPA

It is valuable to note that some patients may present initially as PPA, but other issues are noted. Sometimes this relates to additional non-verbal symptoms given on the history or noted during cognitive testing. Since the label PPA implies isolated language problems, we use “PPA+” to distinguish persons with multidomain impairment but with language as the most prominent deficit. One may still distinguish distinct PPA aphasia subtypes to predict underlying pathology and help direct subsequent testing. In some cases, there may be patients with pure language deficits blending features of multiple subtypes, which can be referred to as a mixed PPA [13]. Other times the language features do not clearly fit a subtype and remain unclassifiable, though some groups are attempting further classification schemes [21, 22] (Table 1). 

**Table 1:** Characteristic features of PPA subtypes

Syndrome	Agrammatic/Nonfluent PPA	Semantic PPA	Logopenic PPA
Features	• Grammatical errors in speech/writing • Difficulty Comprehending Complex Non-Canonical Grammar • Labored speech with speech apraxia	• Impaired single-word comprehension • Impaired naming • Semantic Paraphasic Errors • Surface dyslexia	• Impaired word retrieval • Impaired repetition • Phonemic Paraphasic Errors

### Clinical Testing

A clinician should direct testing to achieve two sequential goals: identifying first the PPA syndrome and then the underlying pathology. Directing testing for underlying pathology depends on identifying the PPA syndrome.

1. ***Cognitive Testing***

**Fig. 2:**
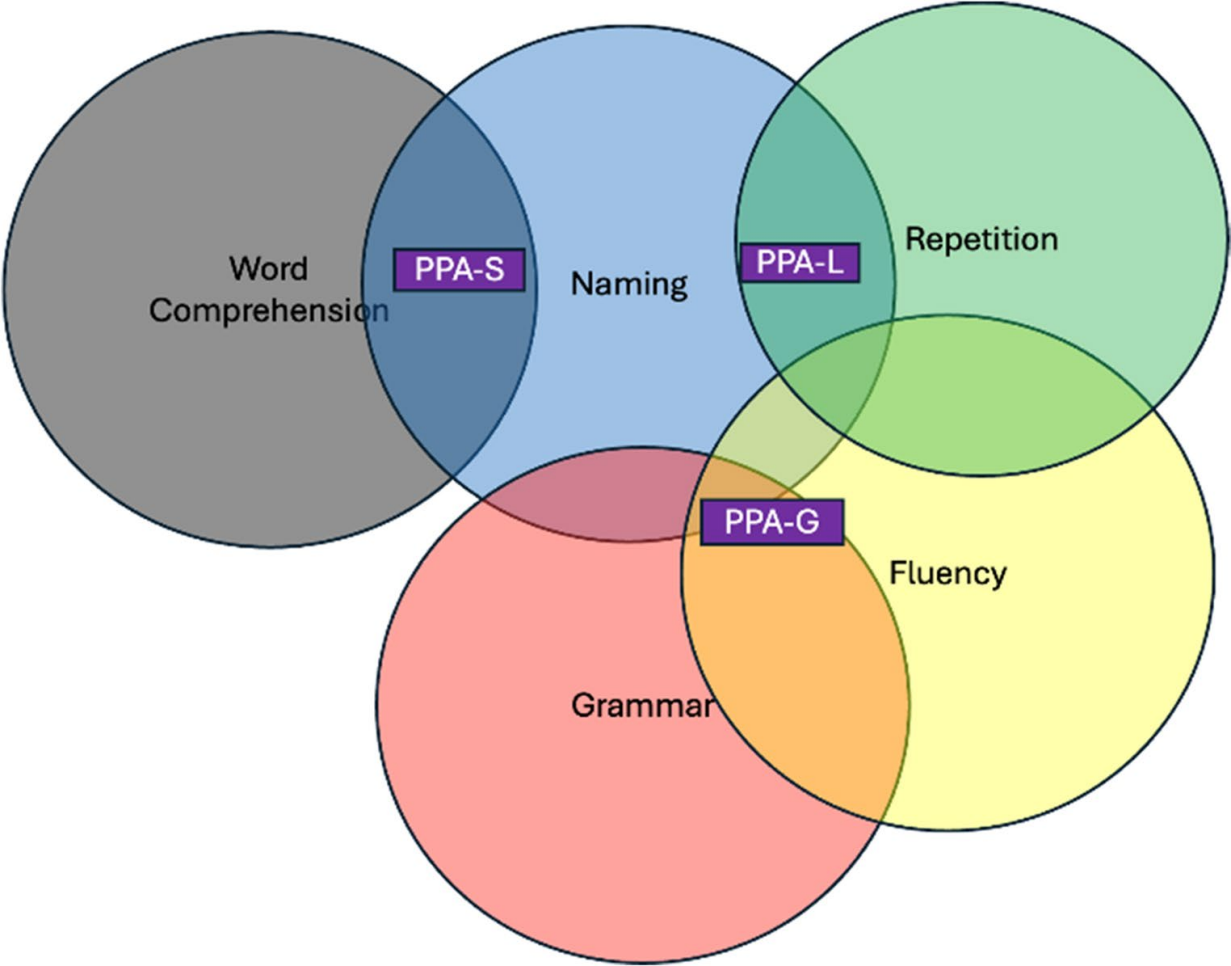
Ven diagram of the major clinical features of the three main PPA subtypes

A thorough cognitive exam is essential for determining whether a patient has PPA and, if so, which type. Many exams can be done at the bedside by a neurologist familiar with a brief repertoire of maneuvers. The exam should interrogate domains outside of language, like processing speed or visual-spatial deficits, that could give the false appearance of naming or other language difficulty. This is admittedly challenging, especially considering many tests are dependent on language, even if only for instructions. A neuropsychological evaluation can be particularly helpful if there is a question of impairment in non-language domains, thus distinguishing PPA from PPA+. A comprehensive cognitive exam can be read about in more detail elsewhere [23, 24]. A formal bedside language exam will be outlined here and focuses on *spontaneous speech*,* comprehension*,* confrontation naming*,* fluency*,* repetition*,* reading*,* writing*,* and motor speech* (Fig. 2).

### Spontaneous Speech

Before administering specific tests, the language exam begins while taking a history. One should observe speech for circumlocutions, word finding difficulties, paraphasic errors (mispronunciations or incorrect word choice), fluency, pronunciation, and prosody (intonation and emphasis). One can also use prompts such as the classic Cookie Theft Picture for a dedicated observation of spontaneous speech.

### Comprehension

One can get some sense of comprehension during the interview by observing how individuals respond to questions. Formal comprehension often includes asking the patient to describe common (e.g. “airplane”) and less common (e.g. “thimble”) words. You can instruct the patient to “point to the source of illumination in the room”, to see if the less common word “illumination” is received. The patient should point to the light or window. Our clinic also often uses a set of objects and toy animals, asking patients to match a word with an object. This tests comprehension of single words which, as noted above, is a hallmark of PPA-S.

Comprehension of grammar is impaired in PPA-G and can be tested using non-canonical grammar transformations that retain object-subject relationships to canonical forms. We often use a picture prompt from the Northwestern Assessment of Verbs and Sentences where patients can point to pictures of dogs and cats chasing each other. The patient is given the picture and asked to point to “the cat that is being bitten by a dog”(non-canonical) versus “the dog biting the cat”(canonical) [12]. 

### Confrontation Naming

Naming typically includes showing objects to individuals who are asked to say the name. One can use an array of household objects and toy animals for informal testing. Formalized exams like the Boston Naming Test have norms that can objectively measure naming and be followed longitudinally [25, 26]. For patients with visual impairment or for use on telephone- based exams, a verbal naming task can be used [27]. One should be careful to understand whether the naming difficulty is “one-way”, with difficulty only in word generation as opposed to a “two-way” naming issue in PPA-S where word generation and comprehension of the objects name are impaired. This can be assessed by giving multiple-choice options for an object they cannot name; if the naming issue is “two-way”, the options will not help.

### Fluency

Fluency can be measured through assessing the number of words produced per minute in spontaneous speech. The average fluency is around 130+/−20 in cognitively normal adults. Counting individual words during a history taking is often impractical but an experienced examiner will develop a general sense. One could also use standardized prompts like the classic Cookie Theft picture to assess [28]. A separate fluency test is asking patients to generate lists of words either in a given category(e.g. animals or clothing items) or for any word starting with a given letter(typically FAS). This is referred to as semantic and lexical fluency, respectively. The average scores are 18 +/−5 words for semantic/category fluency and 14+/−2 for lexical/letter fluency [29, 30]. 

### Repetition

Repetition can be tested by offering sentences to repeat. Prompts given by the NIH Stroke Scale [31]such as “I heard him speak on the radio last night” or from other tests, or even a simple ad-hoc phrase developed by a clinician, are appropriate. The difficulty increases with longer sentences and one should be careful that there is not a problem with working memory or attention.

### Reading/Writing

Reading and writing are often impacted in PPA and not seen in pure speech apraxia. A patient with pure primary progressive *apraxia* of speech will have no problems understanding or producing written language. Reading is tested through supplying a passage or a written command. Patients with language dysfunction may be susceptible to misspellings, use of vague non-specific verbiage, grammatical and punctuation errors when writing [32]. Again, the Cookie Theft Picture serves as a helpful prompt. Patients should be instructed to include correct grammar, punctuation, and spelling.

### Motor Speech

**Fig. 3:**
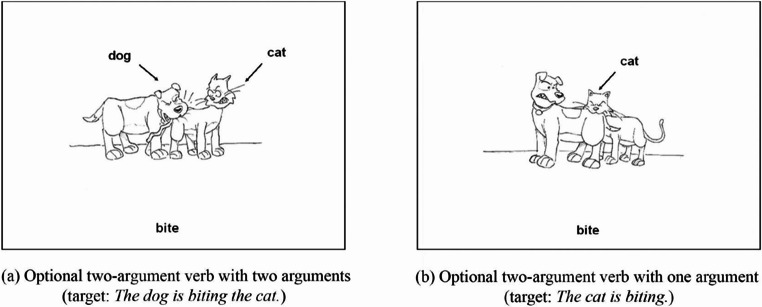
Example picture prompt used for grammar comprehension in the Northwestern Assessment of Verbs and Sentences (NAVS), with permission from Northwestern University and Dr. Cynthia Thompson

Differentiating motor speech problems from language problems can be challenging, but there are several bedside maneuvers that can be used to assess whether a speech apraxia exists. We commonly employ the “Pa-Ta-Ka” tests, where the examiner first has the patient say each syllable individually repeatedly (e.g. “pa-pa-pa”) to assure there is no lingual, guttural, or buccal dysarthria. Then the syllables are combined to test the patient’s ability to create a motor plan to articulate “Pa-Ta-Ka” repeatedly. Also, one can test repetition for complex multisyllabic words like “Methodist Episcopal” or “Turkish constabulary” to look for either inability to repeat such terms. One should also be observing for abnormal length or stress of syllables in speech. A patient with a orobuccal apraxia- beyond speech- may not be able to whistle, mime blowing out a match, or cough on command for an examiner [33] (Fig. 3). 

2. ***Diagnostic Testing***

Each PPA subtype is associated with underlying pathology in a probabilistic fashion. PPA-S is the most specific form, with nearly all cases attributable to FTLD-TDP type C. A small minority of cases are caused by Pick’s disease(a form of FTLD-Tau). PPA-G is typically caused by FTLD-Tau, particularly Corticobasal Degeneration and Progressive Supranuclear Palsy. Rarely, Alzheimer’s disease or FTLD-TDP(type A) may cause PPA-G, constituting around 10% and 20% of cases in our brain bank, respectively. PPA-L is most often due to Alzheimer’s disease, though FTLD tau or TDP accounts for around 40% of cases [34, 35]. Before a diagnosis of PPA is even made, imaging is needed to rule out non-neurodegenerative causes.

#### MRI

MRI is the preferred initial imaging method to rule out stroke, tumor, and other lesional causes of aphasia. Atrophy patterns are also helpful. Each PPA subtype has characteristic patterns of volume loss preferentially impacting areas critical to language functions as described above [36, 37].

PPA-G is associated with atrophy approximately in the inferior frontal gyrus(approximately Broca’s area), PPA-L in the superior temporal gyrus/temporoparietal junction(approximately Wernicke’s area), and PPA-S in the dominant temporal pole. This predilection for the anterior temporal neocortex, distinct from the more typical hippocampal(limbic cortex) atrophy seen in AD and bvFTD. In some cases of TDP-C, we have observed the temporal pole atrophy is severe with T2/FLAIR hyperintense signal indicating gliosis [11, 13]. This is sometimes mistaken for stroke or sequelae of head trauma.

#### FDG-PET

**Fig. 4:**
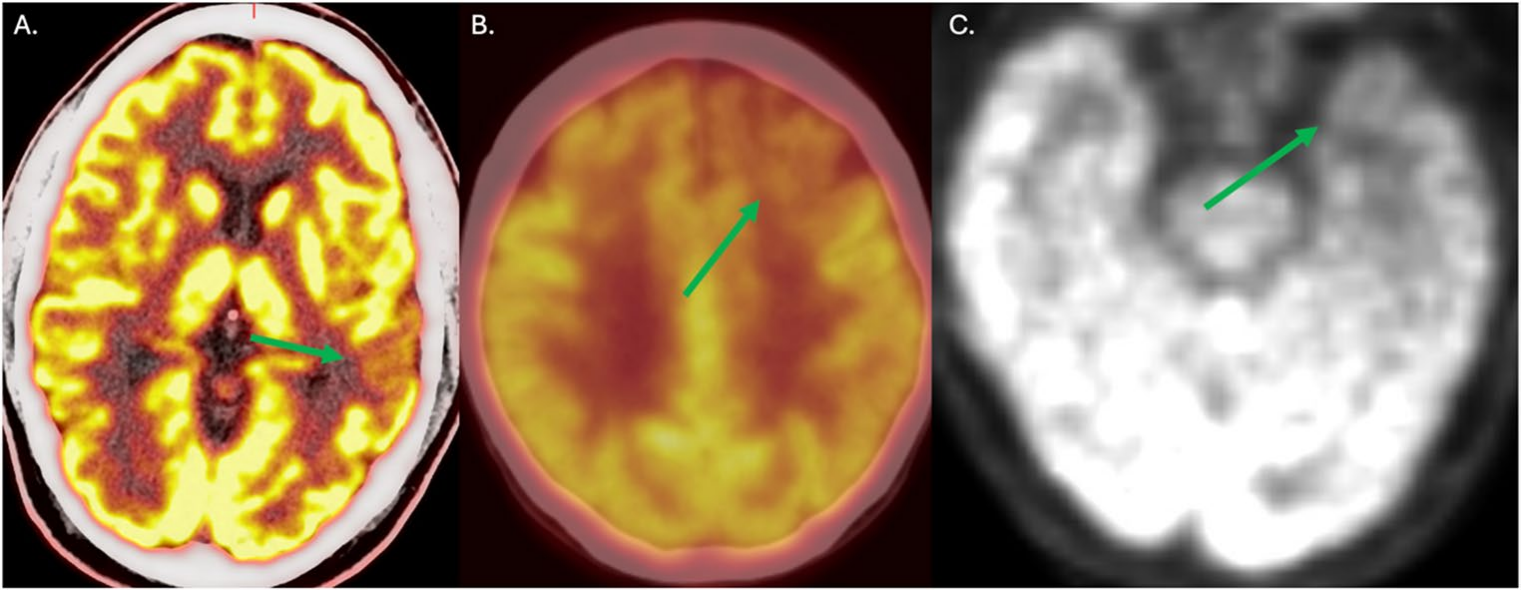
Three patterns of hypometabolism on Flourodeoxy-Glucose Positron Emission Tomography(FDG-PET) in the dominant hemisphere of patients in Northwestern’s longitudinal PPA study with (**A**) PPA-L, (**B**) PPA-G, and (**C**) PPA-S correspond to the anatomy in Fig. 1

A classic functional imaging method in the diagnosis of PPA is a metabolic PET scan using fluorodeoxyglucose(FDG). As with atrophy patterns following regions of interest for each PPA syndrome, a similar pattern of hypometabolism may be seen (Fig. 2). The dominant L inferior frontal gyrus, superior temporoparietal junction, and anterior temporal pole in PPA-G, L, and S respectively. One should be cautious as FDG PET in very mild cases as it is insensitive to early changes (Fig. 4).

#### Biomarkers of Neurodegeneration

Separate from structural and functional imaging, fluid biomarkers stand as a central tool in the diagnosis of AD in the setting of PPA. Currently, we can only reliably measure the AD biomarkers using clinically available assays. The most established biomarkers are spinal fluid biomarkers and amyloid PET imaging.

Spinal fluid biomarkers will give a level of amyloid-beta 42, total-tau(a non-specific marker of neurodegeneration), and an AD-specific isoform of phospho-tau. An Amyloid-PET scan uses a radionucleotide tracer to bind amyloid plaques in the brain, resulting in loss of signal differentiation between cortex and white matter imaging. This is FDA-approved based on visual determination, though software packages can quantify tracer binding, providing precise data for research. In general, CSF and amyloid PET are felt to be similarly accurate [38] when compared to each other, though comparison to final pathology at autopsy is lacking [39]. A tau-PET tracer for AD is FDA approved and potentially prognostic for novel anti-amyloid therapy, but is inaccessible outside of research settings [40]. 

Much attention has been given to blood-based biomarkers in the last few years. This represents a major advancement in the biomarker field and may revolutionize early detection and treatment monitoring. There is particular interest in P-Tau 217. This form of tau is highly sensitive for predicting positive amyloid PET scan [41]. Given the accessibility and affordability compared to CSF and PET, P-Tau 217 has improved enrollment procedures for clinical trials. However, around 25% of patients failed secondary confirmation with PET imaging in the AHEAD study [42]. There is an ever-expanding array of companies offering this test; however, there is significant variability in accuracy [43]. Thus, blood tests are not yet a routine practice and should be used with attention to the specific assay and patient.

Each biomarker discussed can detect AD, particularly Aβ, in pre-symptomatic stages. For this reason, one should not interpret biomarker results in isolation and should use the clinical context. Particularly in the case of PPA-S, where TDP-C is so prevalent, a positive AD biomarker test should be met with skepticism, as previously reported in a clinical-pathological correlation in a case report [44] and observed in our own clinical practice.

#### Genetics

Genetic testing is another important diagnostic consideration. 10–20% of FTD may be due to a genetic cause [45] and genetic testing can be offered after Alzheimer’s disease has been ruled out. In FTLD, the most common mutation is in C9orf72. PPA is different, with the GRN mutation having the strongest association. Of GRN carriers with PPA, it is often a PPA-G phenotype, though presentations outside the typical classification schemes may also occur [46]. PPA is not associated with APOE genotype or other genetic causes of AD.

### Treatment

Treatment for PPA, unfortunately, like most neurodegenerative conditions, is limited. Regardless, clinicians have options of non-pharmacological and pharmacological approaches.

#### Non-pharmacological

Making a diagnosis of PPA indicates preserved faculties like memory, visual-spatial abilities, judgment, and insight. Educating patients and families on preserving other cognitive faculties may help maintain independence and improve quality of life. A formal neuropsychological evaluation may give a clinician added confidence that other cognitive domains are preserved. Driving, shopping, financial management, and even working can often continue early on. In the case of a pure motor-speech problem, the patient and family should be educated that writing instead of speaking may bypass much of their difficulties.

Regardless, PPA poses unique and isolating challenges. Participation in support groups can provide an important source of solidarity and emotional support when facing PPA. Patients/caregivers may also help those impacted develop their own compensatory strategies, applying insights from others. Our center has developed a multidisciplinary program tailored to the social/emotional needs of this population [47]. 

Additional adaptive or non-pharmacologic strategies may be tailored to the patient’s needs through speech-language therapy (SLT). Subtyping PPA may help therapists tailor their strategies, though they typically reconfirm. SLT uses two broad approaches: **impairment-based treatments** (which attempt to improve language functions) and **compensatory treatments** (which develop strategies to work around one’s difficulties). Not all speech-language pathologists have experience in treating PPA, and patients should seek therapists familiar with these approaches.

Impairment-based strategies often focus on **word-retrieval interventions**, often using phonemic or semantic cues to aid naming of pictures or objects. Improvements in anomia have been seen across subtypes using this approach in a comprehensive review [48]. This can be tailored to patient’s areas of weakness, with PPA-L practicing more phonemic cueing and PPA-S more semantic cueing. The improvements may be temporary and with limited generalizability to non-practiced words, however. **Script training** is another approach focusing on commonly used phrases and situations to prepare for real-world situations. Such script training may be durable with evidence of retained scripts up to 12 months [49]. There are also programs described to assist in motor speech problems [50]such as oral reading tasks, but studies are limited.

Compensatory treatments typically focus on low-tech solutions like gestures, flashcards, or picture aides to assist in communication. Patients may use small, laminated pictures on a key ring and a comprehensive binder can be created and updated based on patients/families’ observations of difficult words/topics [51]. For comprehension-based difficulties in PPA-S, the family can be coached to speak more slowly, provide additional gestures or descriptors. Likewise, family can help by cueing patients to talk around words that are missing or offer the first letter of the word, so family can help with retrieval. Technology, has made SLP more accessible with successful implementation of telehealth SLP directed towards PPA [52]. 

Recently, a working group developed unified outcome constructs for communication interventions identifying: “(1) Participate in conversations with family and friends, (2) get words out, (3) be more fluent, (4) convey a message by any means, and (5) understand what others are saying” [53]. No matter what the approach, re-evaluation on a yearly basis is recommended. If there is any sign of decline, the patient can be referred to SLT again for re-evaluation and treatment. In severe stages of the disease where language impairment is too severe, the goal is usually to work with care partners to facilitate communication.

**Fig. 5:**
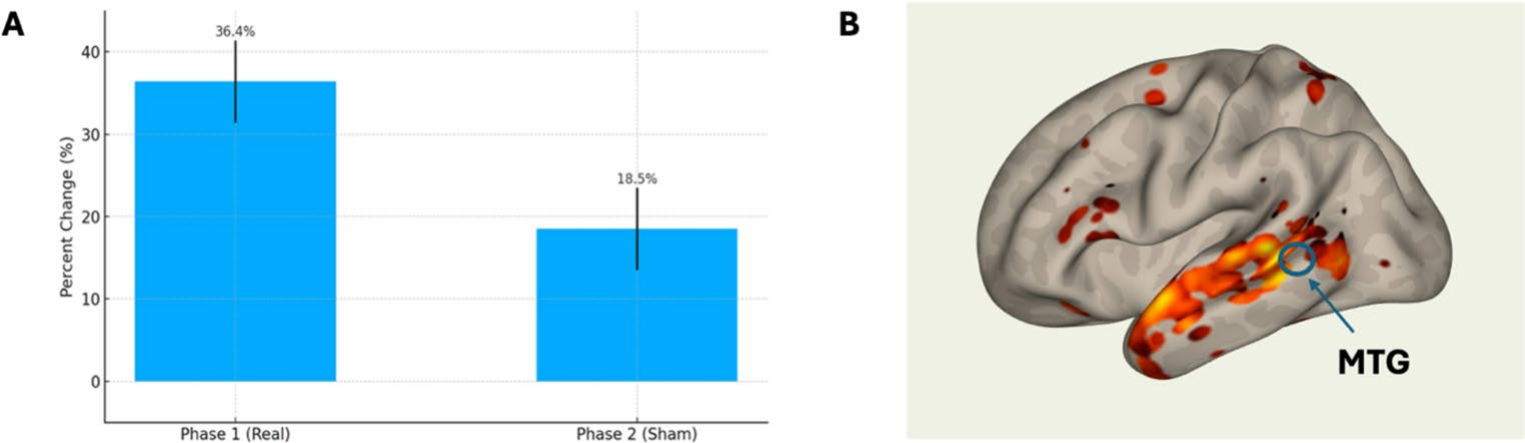
Example of improvement with combined lexical retrieval therapy(LRT) and tDCS intervention. A: % change in object naming in real (phase 1) vs. sham tDCS (phase 2) in combination with LRT. B: resting state functional connectivity demonstrating increased connectivity between posterior middle temporal gyrus (MTG), where tDCS was applied (blue ring), lateral and anterior temporal lobe which are involved in the process of naming

Several trials have been done looking at neuromodulation using mostly transcranial direct current stimulation(tDCS) and some transcranial magnetic stimulation(TMS), often in combination with an SLP intervention. In a recent Cochrane review, there were a total of 10 studies identified on 132 patients [54]. There is a suggestion of moderate benefit but current studies are small and more study is needed. Future investigations will focus on choosing the right-hemisphere network stimulation combined with evidence of target engagement. Figure 5 shows LRT was used in an individual with logopenic PPA due to Alzheimer’s disease. The patient showed improvement in picture naming following 16 sessions of combined LRT and high definition tDCS applied to posterior middle temporal gyrus (pMTG). Target engagement was confirmed using functional MRI resting connectivity analysis.

#### Pharmacological Strategies

Medication trials have investigated efficacy of bromocriptine [55]steroids [56]and memantine [57, 58] have been tried without benefit. While non-pharmacologic approaches are directed at symptoms, the authors’ approach to pharmacologic treatment in PPA is directed at etiology. Despite much effort, there are no pharmacological treatments for frontotemporal dementia, including FTLD-PPA. Selecting disease-specific therapy is, dependent on ruling in AD.

In AD, there is an increasing array of treatment options. This consists of cholinesterase inhibitors(CIs), like donepezil, which are found to be effective in all stages of AD. And memantine is most beneficial in the moderate to severe stages [59]. While this approach is extrapolated from other AD trials [60, 61]there are mixed results in existing studies on PPA [57, 58, 62]. However, there is direct evidence of cholinergic dysfunction in PPA due to AD, providing a biological basis for CI use [63]. Also, a recent retrospective study demonstrated similar results using CIs when compared to amnestic AD [62]. Most recently two anti-amyloid monoclonal antibodies, donanemab and lecanemab, were approved by the FDA for treatment of early-stage AD [64, 65]. The drugs slow the progression of mild AD but require careful consideration before initiation and monitoring during treatment due to potential side effects. While the trials only included amnestic AD, it has been our practice to discuss this nuance with PPA patients but not exclude patients solely based on their atypical symptoms (Table 2).

These medications are not effective in FTLD and their use is not recommended. Research for FTLD treatment is ongoing, including a trial specifically on PPA-S using verdiperstat, a myeloperoxidase inhibitor hypothesized to benefit TDP-43 proteinopathy [66].

**Table 2:** PPA treatment overview

Category	Intervention	Examples	Note
Speech Language Therapy			Increased accessibility through telemedicine
	Impairment based	Script training, word retrieval interventions	
	Compensatory based	Picture aides, cueing, caregiver training	
Pharmacological			
	Cholinesterase inhibitors	Donepezil, galantamine, rivastigmine.	In AD patients
	NMDA Receptor antagonists	Memantine	Moderate to severe AD dementia
	Anti-Amyloid Monoclonal Antibodies	Lecanemab, Donanemab	MCI or Mild Dementia due to AD
Neuromodulation			
	Transcranial Magnetic/Direct Current Stimulation(TMS/TDCS)	Currently experimental, available through research	Usually paired with Speech Language Therapy. Techniques undergoing refinement.

## Conclusions

Through familiarity with PPA, its subtypes, and the subtypes’ associations with underlying etiology, one can direct workup and treatment. Making a correct diagnosis also provides closure to families and patients. Clinicians have an increasing array of tools at their disposal for treatment if due to AD and research is ongoing. Regardless, PPA remains a progressive, incurable, degenerative illness, and more research is needed. The increasing availability of biomarker testing and telemedicine, hopefully, will allow more patients to access accurate diagnosis and expert SLT services.

## Data Availability

Data provided for figure 5 is preliminary, previously unpublished data from an ongoing study. As this is a review paper, we will not provide data for sharing unless necessary.
